# Dr. William H. Robb

**Published:** 1898-02

**Authors:** 


Dr. William H. Robb, of Amsterdam, N. Y., died at Selma, Alabama, whither he had gone for his health, January 12, 1898, aged 54 years. He had practised many years at Amsterdam, where he was esteemed as a good physician and prominent citizen.

